# NRF2 Alters Mitochondrial Gene Expression in Neonate Mice Exposed to Hyperoxia

**DOI:** 10.3390/antiox11040760

**Published:** 2022-04-11

**Authors:** Heather L. Vellers, Hye-Youn Cho, Wesley Gladwell, Kevin Gerrish, Janine H. Santos, Gaston Ofman, Laura Miller-DeGraff, T. Beth Mahler, Steven R. Kleeberger

**Affiliations:** 1 Health and Exercise Science Department, University of Oklahoma, Norman, OK 73019, USA; 2 Immunity, Inflammation, and Disease Laboratory, National Institute of Environmental Health Sciences, Research Triangle Park, NC 27709, USA; cho2@niehs.nih.gov (H.-Y.C.); miller12@niehs.nih.gov (L.M.-D.); kleeber1@niehs.nih.gov (S.R.K.); 3 Molecular Genomics Core Laboratory, National Institute of Environmental Health Sciences, Research Triangle Park, NC 27709, USA; gladwell@niehs.nih.gov (W.G.); gerrish@niehs.nih.gov (K.G.); 4 Division of the National Toxicology Program, Mechanistic Toxicology Branch, National Institute of Environmental Health Sciences, Research Triangle Park, NC 27709, USA; janine.santos@nih.gov; 5 Department of Pediatrics, The University of Oklahoma Health Sciences Center, Oklahoma City, OK 73104, USA; gaston-ofman@ouhsc.edu; 6 Division of the National Toxicology Program, Comparative and Molecular Pathogenesis Branch, Research Triangle Park, NC 27709, USA; mahler1@niehs.nih.gov

**Keywords:** mitochondrial sequencing, mtDNA copy number, heteroplasmy, mitochondrial function, NRF2, DNA lesions

## Abstract

Approximately 1 in 10 newborns are born preterm and require supplemental oxygen (O_2_) in an extrauterine environment following birth. Supplemental O_2_ can induce oxidative stress that can impair mitochondrial function, resulting in lung injury and increased risk in early life pulmonary diseases. The nuclear factor-erythroid 2 related factor 2 (NRF2) protects the cells from oxidative stress by regulating the expression of genes containing antioxidant response elements and many mitochondrial-associated genes. In this study, we compared *Nrf2*-deficient (*Nrf2^−/−^*) and wild-type (*Nrf2^+/+^*) mice to define the role of NRF2 in lung mitochondrial genomic features in late embryonic development in mice (embryonic days, E13.5 and E18.5) versus birth (postnatal day 0, PND0). We also determined whether NRF2 protects lung mitochondrial genome parameters in postnatal mice exposed to a 72 h hyperoxia environment. We found *Nrf2^−/−^* embryonic lungs were characterized by decreases in mtDNA copies from E13.5 to E18.5. Interestingly, *Nrf2^−/−^* heteroplasmy frequency was significantly higher than *Nrf2^+/+^* at E18.5, though this effect reversed at PND0. In postnatal mice exposed to hyperoxia, we identified three- to four-fold increases in mitochondria-encoded mitochondrial genes, which regulate oxidative phosphorylation. Overall, our findings demonstrate a potentially critical role of NRF2 in mediating long-term effects of hyperoxia on mitochondrial function.

## 1. Introduction

Approximately 1 in 10 newborns are born preterm and require supplemental oxygen (O_2_) in an extrauterine environment following birth [1,2]. While supplemental O_2_ is widely used in neonatal care, there remain ongoing challenges in establishing optimal O_2_saturation delivery to avoid detriments associated with too much or too little O_2_. Oxidative stress and associated systemic inflammatory responses underlie lung and other organ injuries with too-high supplemental O_2_. Because of the continual challenge to develop and implement optimal O_2_ supplementation in neonates that require it, there is a critical need to identify effective therapeutics to inhibit severe oxidative-stress-induced tissue injury and disease development in the lung and other affected tissues (e.g., heart).

Oxidative stress can impair mitochondrial function at the cellular level, leading to the organelle increasing its production of reactive oxygen species (ROS), which further challenges the cell’s antioxidant capacity. The nuclear factor-erythroid 2-related factor 2 (NRF2), a ubiquitous master transcription factor, protects the cells from oxidative stress by regulating the expression of genes containing antioxidant response elements (ARE), including antioxidant enzymes and many mitochondrial-associated genes. By activating NRF2, cells dampen oxidative stress and the associated damage [1]. Previous works by our group showed that NRF2 protects neonate mice from O_2_ exposure [3,4], and prenatal supplementation of a NRF2 agonist, sulforaphane, mitigated neonatal lung injury from O_2_ exposure at birth [5]. The mechanism of sulforaphane pulmonary protection was associated with increased transcriptomic mitochondrial energy metabolism in adult mice [6]. Significant evidence demonstrates that NRF2-mediated nuclear regulation on mitochondrial function occurs via ARE modulated transcriptional activation of mitochondrial antioxidant and bioenergetic pathways [6,7,8,9]. Thus, targeting NRF2-ARE to enhance cellular antioxidant stress appears effective in protecting against lung injury with O_2_ exposure at birth. However, whether this effect of NRF2 involves regulation of mitochondrial function and its genome or other cellular targets remains unclear.

Mitochondrial DNA (mtDNA) encodes 37 genes, including 13 that are protein-coding and responsible for expression of critical subunits of the electron transport chain (ETC), and thus energy production through the process of oxidative phosphorylation. The mtDNA is highly susceptible to damage and mutations given its close proximity to the ETC and the lack of histone protection [10]. Consequently, mtDNA damage can impair mitochondrial function by interfering with transcription/replication of the mtDNA or by leading to mutations [11]. In addition, it has become increasingly clear that changes to mtDNA and the associated effects on mitochondrial metabolism significantly impact signaling, the epigenome and gene expression [12]. Analyzing mtDNA features in a mouse model without antioxidant properties (*Nrf2* deficient) to protect mitochondria against O_2_-induced damage could highlight important epigenetic regulation of the nuclear genome, arising from NRF2, in mitochondrial biology.

Embryonic development is a critical period that prepares the fetus to transition from a hypoxic environment in the womb (1–9% O_2_), to one that is higher O_2_ (normoxia, 21% O_2_) at birth [13,14]. During the early stages (embryonic day 1, E1–E13), cells are heavily reliant on glycolysis, but they transition into greater usage of oxidative pathways to produce adenosine triphosphate (ATP) during late gestation and birth [15]. Along with this metabolic shift, mitochondria undergo significant stages of maturation (e.g., developing cristae), making them highly susceptible to damage with high ROS production during early embryonic days, when glycolysis is primarily used for ATP production, and late gestation, when oxidative phosphorylation is used for ATP generation. Thus, given that NRF2 serves as a critical player in regulating antioxidant mechanisms to prevent mitochondrial and/or tissue injury caused by excessive ROS production, it is important to also consider the role of NRF2 during embryonic development and birth to determine if it impacts postnatal outcomes in metabolism.

In this study, we used an *Nrf2* knockout mouse model to define the role of NRF2 on lung mitochondria in late embryonic development in mice (embryonic days, E13.5 and E18.5) versus birth (postnatal day 0, PND0). We also determined whether NRF2 protects lung mitochondria in postnatal offspring exposed to a 72 h hyperoxia environment (100% O_2_). In this work, we elected to assess the role of NRF2 on postnatal development at 5 weeks of age, given this time period immediately follows the weaning period, just before puberty begins [16]. Given that evidence suggests that NRF2 is responsible for facilitating antioxidant properties to protect mitochondria when exposed to oxidative stress [17], we hypothesized that our findings would indicate significant alterations in mitochondrial genomic parameters.

Our major findings included reduced expression of several mitochondrially-encoded genes, includingNADH dehydrogenase 1 (*mt-Nd1*), *mt-Nd4*, *mt-Nd6*, cytochrome c oxidase 1 (*mt-Co1*), and cytochrome b (*mt-Cytb*) during embryonic development (E13.5 to E18.5). We only observed an effect of NRF2 during this time in *mt-Nd4*, where *Nrf2^−/−^* embryonic lung gene expression was significantly lower than *Nrf2^+/+^*. Next, we found that nuclear DNA (nDNA) lesions were significantly increased in *Nrf2^−/−^* embryonic lungs from E13.5 to E18.5, but not in the wild type. While we did not observe changes in mtDNA integrity during embryonic development or at birth (PND0), mtDNA copy number decreased from E13.5 to E18.5. Lastly, at E18.5, we found significantly higher mtDNA heteroplasmy frequency in *Nrf2^−/−^* embryonic lungs than *Nrf2^+/+^*. Interestingly, this effect reversed at PND0, where heteroplasmy was markedly lower in *Nrf2^−/−^* lungs. Collectively, these findings highlight a potential role of NRF2 in metabolic adaptations during embryonic development.

During postnatal development and exposure to hyperoxia, we identified significant increases in gene expression for all the mtDNA-e-encoded that we selected (*mt-Nd1*, *mt-Nd4*, *mt-Nd6*, *mt-Co1*, and *mt-Cytb*). Unlike embryonic development, nDNA lesions, mtDNA copy number and heteroplasmy were unaltered. However, at PND4, *Nrf2^−/−^* mice had significantly higher mtDNA lesions, though they remained at a similar level until the end of the study (i.e., 5 weeks) and were unaffected by exposure.

## 2. Methods and Materials

### 2.1. Animals

Male, 6–8-week-old *Nrf2^+/+^* (ICR) and *Nrf2^−/−^* (ICR.129P2-*Nfe2l2^tm 1Mym^*) mice produced from breeding colonies at the National Institute of Environmental Health Science (NIEHS) were used in the current study. For aim 1 (assessment of the role of NRF2 on developmental lung mitochondrial genome characteristics), we collected lungs from embryos on E13.5 (*Nrf2^+/+^* embryos: *n* = 5, *Nrf2^−/−^* embryos: *n* = 5), E18.5 (*Nrf2^+/+^* embryos: *n* = 5, *Nrf2^−/−^* embryos: *n* = 4), and PND0 (*Nrf2^+/+^* embryos: *n* = 5, *Nrf2^−/−^* embryos: *n* = 6). In detail, time-pregnant *Nrf2^+/+^* and *Nrf2^−/−^* dams were euthanatized at E13.5 or E18.5 by carbon dioxide. Each had its abdominal cavity opened, and the uteri were removed intact. The uterine horn was spread out on a petri dish with ice-cold PBS to cut between each sac to remove embryos. Extraembryonic membranes (amnion, yolk sac, chorion, allantois) from each embryo were removed, and the embryo was separated. Embryonic lungs were collected under a dissection microscope.

For aim 2 (determination of the role of NRF2 in postnatal offspring mouse lung protection from hyperoxia), offspring *Nrf2^+/+^* and *Nrf2^−/−^* mice at PND0 were randomly assigned to either hyperoxia (>95% O_2_ for 72 h) in a whole-body chamber or to room air (normoxia) as indicated below and published previously [3]. From these postnatal mice, we collected lungs on PND4 that were either exposed to 72 h hyperoxia (*Nrf2^+/+^* mice: *n* = 5, *Nrf2^−/−^* mice: *n* = 4) or as an air control (*Nrf2^+/+^* mice: *n* = 5, *Nrf2^−/−^* mice: *n* = 4). Lastly, we had another subset of mice that underwent a 5-week recovery period following exposure to hyperoxia (72 h O_2_ + 5-week rec, *Nrf2^+/+^* mice: *n* = 4, 72 h O_2_ + 5-week rec, *Nrf2^−/−^* mice: *n* = 5) or air control (air + 5-week rec, *Nrf2^+/+^* mice: *n* = 3, air + 5-week rec, *Nrf2^−/−^* mice: *n* = 3). Immediately following designated exposure, mice were euthanized by sodium pentobarbital overdose (104 mg/Kg).

In both embryonic and postnatal stages, lungs were harvested at designated times by snap freezing in liquid nitrogen and stored at −80 °C until used for molecular analyses. All animal use was approved by the NIEHS Animal Care and Use Committee.

### 2.2. Neonate Hyperoxia Exposure

Two days before scheduled delivery, time-pregnant *Nrf2^+/+^* and *Nrf2^−/−^* mice were cohabitated with time-pregnant foster dams. Upon birth (PND0), neonatal mice from multiple litters with the same prenatal treatment or same genotype were pooled and randomly reassigned to exposure groups with a foster dam per group. The number of mice per cage was kept at a maximum of 12 and matched between comparison groups to control for the effects of the litter size on nutrition and growth. At 24 h after birth (PND1), neonatal mice were placed in cages and inserted into an inhalation chamber, and exposed to hyperoxia (UHP grade, Min. purity 99.994% O_2_, National Welders, Durham, NC, USA) continuously for 2 or 3 days with their foster dams. During exposure, foster dams had free access to food (modified AIN76A; Harlan Teklad) and water. The temperature (72 ± 3 °F) and humidity (50 ± 15%) of the chamber were monitored, and mice were exposed to a 12 h light–dark cycle. The entire volume of gas in the chamber was flushed via total system flow every 3–4 min to maintain consistent O_2_ concentration and to avoid CO_2_ accumulation. Neonatal mice and their foster dams assigned to room-air controls were placed in cages placed on a countertop with food and water provided ad libitum for the same exposure duration. The hyperoxia chamber was opened briefly after 24 h of exposure to replace foster dams and check animal health (morbidity and mortality). For neonates groups used to assess acute responses to 72 h hyperoxia, *Nrf2^+/+^* and *Nrf2^−/−^* pups were removed from the chamber at the end of a 3-day exposure to hyperoxia or room air, and pups were euthanized by sodium pentobarbital overdose (0.02 mL per neonate). To assess responses following 5 weeks of the 72 h hyperoxia exposure, *Nrf2^+/+^* and *Nrf2^−/−^* pups remained with foster dams until PND14, then were weaned and group housed for 5 weeks, then euthanized.

### 2.3. Lung Extraction and Mitochondria-Encoded Mitochondrial Gene Expression

We isolated total lung RNA with the Maxwell RSC simply RNA Tissue Kit (Promega, Madison, WI, USA). To synthesize cDNA, we used 100 ng with the iScript cDNA synthesis kit (Bio-Rad, Hercules, CA, USA) following the manufacturer’s recommendations. The QX200 Droplet Digital PCR System uses supermix for probe (no dUTP) (Bio-Rad); all the procedures follow the manufacturer’s instructions (Bio-Rad). The PCR reaction mixture was assembled from 2× supermix for probe (no dUTP), probe mix (final 250 nM, for each probe), and 1 μL of the diluted 1–50 cDNA template in a final volume of 25 μL. Twenty microliters of each reaction mix were converted to droplets with the QX200 droplet generator (Bio-Rad). Droplet-partitioned samples were then transferred to a new 96-well plate, sealed with PX1 PCR plate sealer, and cycled in a T100 Thermal Cycler (Bio-Rad) under the following cycling protocol: 95 °C for 10 min (polymerase activation), followed by 40 cycles of 95 °C for 30 s (denaturation) and 60 °C for 1 min (annealing), followed by 98 °C for 10 min (polymerase deactivation) an infinite 4-degree hold. The cycled plate was then transferred and read in the FAM and HEX channels using the QX200 reader (Bio-Rad). The number of template molecules per microliter of material was estimated by Quanta-SoftTMddPCR software (Bio-Rad) and the samples were normalized by an average of Rpl13a for all samples. We selected 5 of the 13 mitochondria-encoded mitochondrial genes that encode for oxidative phosphorylation including *mt-Nd1*, *mt-Nd-4*, *mt-Nd6*, *mt-Co1*, and *mt-Cytb*. For each gene, we divided the concentration of Rpl13a for each sample by the average, which yielded the normalization factor. Following, we then divided the concentration of the mitochondrion gene by normalization factor (i.e., normalized concentration), which we plotted as the normalized concentration (copies/µL). Table 1 provides the mitochondrial gene name, assay ID, and probe fluorophore.

### 2.4. Mouse Mitochondrial DNA Ultra-Deep Sequencing

We employed a long-range PCR (SequalPrep Long PCR Kit, Life Technologies, Grand Island, NY, USA) based method to amplify the complete mitochondrial genome. We used the following overlapping primers to amplify the mtDNA in two halves: Set 1 (10 kb amplicon) with forward (3301, GCC AGC CTG ACC CAT AGC CAT AAT AT), and reverse (13367, GAG AGA TTT TAT GGG TGT AAT GCG G); Set 2 (7.5 kb amplicon) with forward (12791, TCC CAC TCC TAA ACA CAT CC), and reverse (3880, TTT ATG GGG TGA TGT GAG CC). For primer set 1, we used the following PCR conditions: 94 °C for 2 min; 10 cycles: 94 °C for 10 s, 57 °C for 30 s, 68 °C for 10 min; 25 cycles: 94 °C for 10 s, 57 °C for 30 s, 68 °C for 12 min; 72 °C for 5 min (Gene Amp PCR system 9700, Applied Biosystems, Foster City, CA, USA). Then, for set 2, we used the following PCR conditions: 94 °C for 2 min; 10 cycles: 94 °C for 10 s, 51 °C for 30 s, 68 °C for 7.5 min; 25 cycles: 94 °C for 10 s, 51 °C for 30 s, 68 °C for 9 min; 72 °C for 5 min. Lastly, we cleaned the PCR products with the Zymo DNA Clean and Concentrator kit (Zymo, Irvine, CA, USA), then pooled the mtDNA for library preparation.

### 2.5. Nextera XT Mitochondrial DNA Library Preparation

For the mtDNA library preparation, we employed the same protocol as outlined in our previously published work [18]. Briefly, we used 1 ng of mtDNA for each library preparation with the Nextera XT Sample Preparation Kit, according to the manufacturer’s instructions (Illumina, San Diego, CA, USA). Next, we amplified the fragmented mtDNA with a limited-cycle PCR using the Nextera XT Index Kit. We ran the PCR using the following cycling parameters: 72 °C for 3 min, 95 °C for 30 s, 12 cycles of 95 °C for 10 s, 55 °C for 30 s, 72 °C for 30 s, then a final extension of 72 °C for 5 min, and a hold at 10 °C. According to the manufacturer’s instructions, we removed small fragments from the PCR reaction and incubated the sample for 2 min with 90 µL of Agencourt AMPure XP beads (Beckman Coulter, Indianapolis, IN, USA). We then transferred the supernatants to a new microcentrifuge tube and quantified the libraries using the Qubit dsDNA High Sensitivity Kit (Thermo Fisher Scientific, Waltham, MA, USA). We ultra-deep sequenced the libraries on a MiSeq instrument (Illumina) using a 2 × 150 bp paired-end protocol with 20 samples per lane.

Similar to the mtDNA library preparation, the procedures we followed to align mtDNA and call variants adhered to the same guidelines we used in our previously published work [18]. As such, and as previously described, we aligned read pairs using bowtie2, version 2.0.0-beta7 [19], to an index composed of the human mitochondrial genome, acquired from GenBank May 2015 (accession NC_012920). The alignments were performed in “--local” mode using the “--sensitive-local” preset options to allow insertions and deletions relative to the reference, as well as clipping of ends extending beyond the edges of the artificially linearized reference sequence. Fragment lengths of up to 10 kb were allowed, as well as a single mismatch per seed alignment (−X 10,000, −N 1). Variants were identified with a custom script, utilizing a method adapted from Hodgkinson et al. [20] For each sample, depth per allele per strand was determined, allowing a minimum base and alignment quality score of 20. The depth calculation for sites adjacent to or within homopolymer runs considered only reads that traversed the entire repeat. Sites with less than 1000× coverage were not considered, and variants were required to be observed at a frequency of 1% or higher, with a plus to minus strand-coverage ratio greater than 0.1 and less than 0.9. The probability of observing each alternate allele by chance was calculated using a Poisson distribution, with an expected error rate of 0.01 for single-nucleotide polymorphisms (SNPs, derived from the quality score threshold) based on observations reported by Minoche et al. [21]. These *p*-values were adjusted for multiple testing using the Benjamini and Hochberg FDR method, and a significance threshold of 0.05 was applied. The *p*-values were calculated at each position within the mitochondrial genome based on the read counts supporting variants and reference alleles. The global null hypothesis was that no variants were present, and all reads supporting alternate alleles resulted from PCR and sequencing error. The Benjamini and Hochberg FDR method for multiple-testing correction was applied due to the large number of *p*-values calculated, as high as 49,707 for a single sample, if no positions or alternate alleles were filtered. Alternate amino acids were identified for all SNPs based on annotations and protein sequences downloaded from Ensembl, June 2015.

### 2.6. Identification of Informative Mitochondrial DNA Variants and Heteroplasmy

Informative variants were called when we identified a loci such as the one in the mitochondrial genome (16,569 total positions), where mice in a given group differed from other groups according to age, model, and/or exposure. We defined a position with heteroplasmy by the following conditions: a position with at least 1000 read sequencing depth where the alternate allele had one percent or higher occurrence than the reference allele.

### 2.7. Assessment of Lung DNA Lesions and mtDNA Copy Number

DNA was extracted from the whole lung of each mouse using the DNeasy Blood & Tissue Kit (Qiagen, Carlsbad, CA, USA) in accordance with the manufacturer’s instructions. Isolated DNA was quantified with the Qubit™ dsDNA HS Assay Kit on a Qubit Analyzer (Invitrogen, Life Technologies, Grand Island, NY, USA) in triplicate for accurate quantification. All samples were diluted to 3.0 ng DNA/µL in Tris-EDTA (TE) buffer (Promega).

We then used a PCR (qPCR) protocol developed by Santos et al. [22] and Furda et al. [23] that used gene-specific primers to assess for mitochondrial and nuclear DNA lesions, as well as the mtDNA copy number (Table 2), as provided in their protocols for mice.

Briefly, the GeneAmp XL PCR kit was used to prepare the PCRs: 15 ng of total genomic DNA, 1× buffer, 100 ng/μL final concentration of BSA, 200 μM final concentration of dNTPs, 20 pmol of each primer, 1.3 mM final concentration of magnesium, and nuclease-free water to a total volume of 45 μL. For the mtDNA assays only (mtDNA lesions and copy number), a restriction enzyme digest (New England BioLabs Inc., Ipswich, MA, USA) was used in the following conditions to linearize the mtDNA to enable effective amplification per each sample (40 µL total for triplicate analysis): two hours at 37 °C; 3.3 µL nuclease-free water, 5 µL 1× CutSmart^®^ Buffer, 0.5 µL 10× Bovine Serum Albumin, and 1.25 µL HaeII enzyme. Each PCR was started with a “hot start” for two minutes at 75 °C prior to adding the enzyme for the short (SMITO; FailSafe Taq Polymerase; Lucigen, Middleton, WI, USA) and long mitochondrial gene fragment (LMITO; LongAmp Taq DNA Polymerase; New England BioLabs Inc.), and the nuclear gene (β-Pol; LongAmp Taq DNA Polymerase; New England BioLabs Inc.). The qPCR conditions for each primer are provided in the protocol [22,23]. Experimental controls included a non-damaged control (3.0 ng/µL), a non-damaged 50% control (1.5 ng/µL), and no DNA (TE buffer). A PCR tube containing 1× TE instead of DNA (“no template” control) and a PCR tube containing 50% DNA amount (DNA diluted 1:1 first) were used to ensure optimization of the PCR cycles. A fluorescence reading was then obtained using the FL600 Microplate Fluorescence (Bio-Tek; Winooski, VT, USA), and DNA lesions and mitochondrial DNA copy number were calculated as described in the protocol [22,23].

### 2.8. Statistical Analyses

All data are presented as means ± standard error of the mean (S.E.M) for each group. For each DNA lesion and mtDNA copy number assay, experimental sample data are expressed as a relative change compared to non-damaged mouse (C57BL/6J) DNA liver samples.

For aim 1, we employed a two-way ANOVA with time (E13.5, E18.5, PND0) and mouse model (*Nrf2^+/+^* and *Nrf2^−/−^*) as the two factors. For aim 2, we also used a two-way ANOVA with time (72 h O_2_ exposure or air control; or 72 h O_2_ with 5-week rec or air control P4), mouse model (*Nrf2^+/+^* and *Nrf2^−/−^*), and exposure (hyperoxia or normoxia) as the factors. For both aims, we assessed five mtDNA-encoded genes, DNA lesions (mitochondrial and nuclear), and the mtDNA copy number as the independent variables. We used Tukey’s post hoc (α-level: *p* < 0.05). All statistical analyses were performed using JMP Pro 14.0.0. (SAS Institute Inc., Cary, NC, USA).

## 3. Results

### 3.1. Embryonic Development

**Figure 1:**
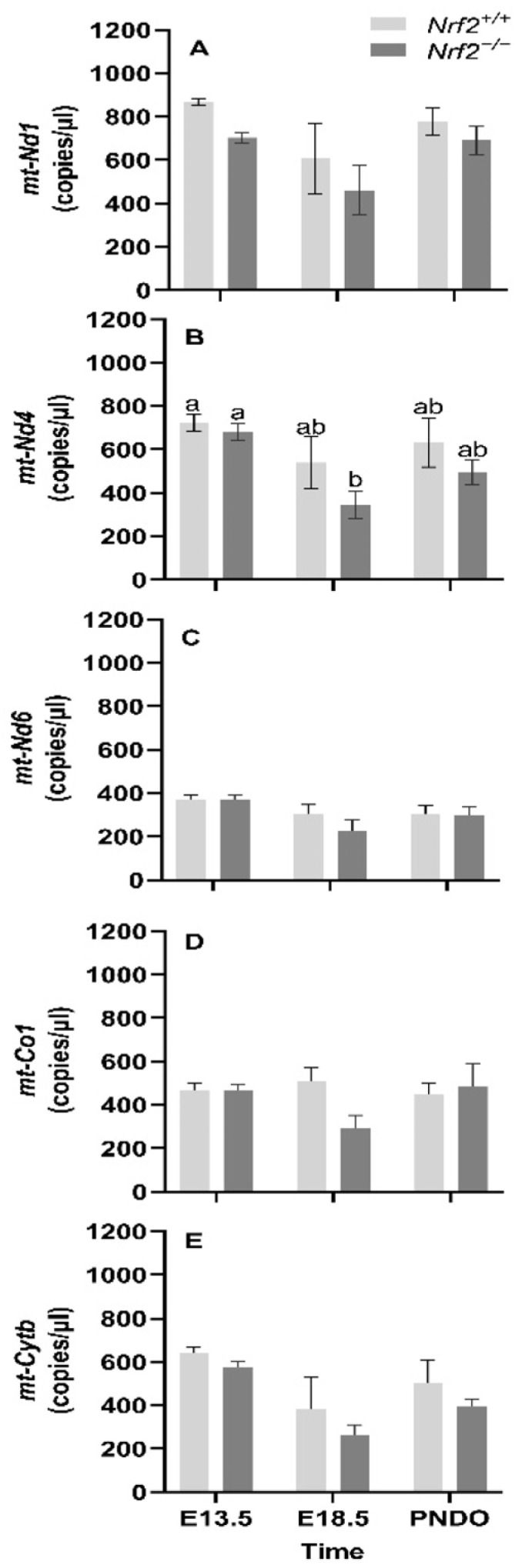
Effect of *Nrf2* deletion on lung mitochondria-encoded mitochondrial gene expression during embryonic development and at birth. Mitochondria-encoded mitochondrial genes, NADH dehydrogenase 1 (*mt-Nd1*) (**A**), *mt-Nd4* (**B**), *mt-Nd6* (**C**), cytochrome c oxidase subunit 1 (*mt-Co1*) (**D**), and cytochrome B (*mt-Cytb*) (**E**), were determined in lungs at embryonic (E) days 13.5 and 18.5 or postnatal day 0 (PND0, day of birth) from wild-type (*Nrf2^+/+^*) and *Nrf2*-deficient (*Nrf2^−/−^*) mice by digital droplet PCR. *Nrf2^+/+^* Mean ± S.E.M. presented (*n* = 5–6/group). A two-way ANOVA was employed with age (E13.5, E18.5, PND0) and genotype (*Nrf2^+/+^*, *Nrf2^−/−^*) as the factors. Letters were used when there were significant main effects (*p* < 0.05). Bars not connected by the same letter are significantly different (*p* < 0.05).

*mt-Nd1, mt-Nd4, mt-Nd6,* and *mt-Cytb* gene expression decreases from embryonic days E13.5 to E18.5: We found that the mean gene expression in *mt-Nd1* (*p* = 0.02; Figure 1A), *mt-Nd6* (*p* = 0.02; Figure 1C), and *mt-Cytb* (*p* = 0.02; Figure 1D) decrease from embryonic day E13.5 to E18.5. We only found a change in gene expression in *Nrf2^−/−^* embryonic lungs in *mt-Nd4* (*p* = 0.03; Figure 1B), which decreased from E13.5 to E18.5 (E13.5: 680 ± 39 *mt-Nd4* copies/µL, E18.5: 334 ± 65 *mt-Nd4* copies/µL; *p* = 0.01); no change was observed in *Nrf2^+/+^* (E13.5: 721 ± 39 *mt-Nd4* copies/µL, E18.5: 539 ± 119 *mt-Nd4* copies/µL).

**Figure 2:**
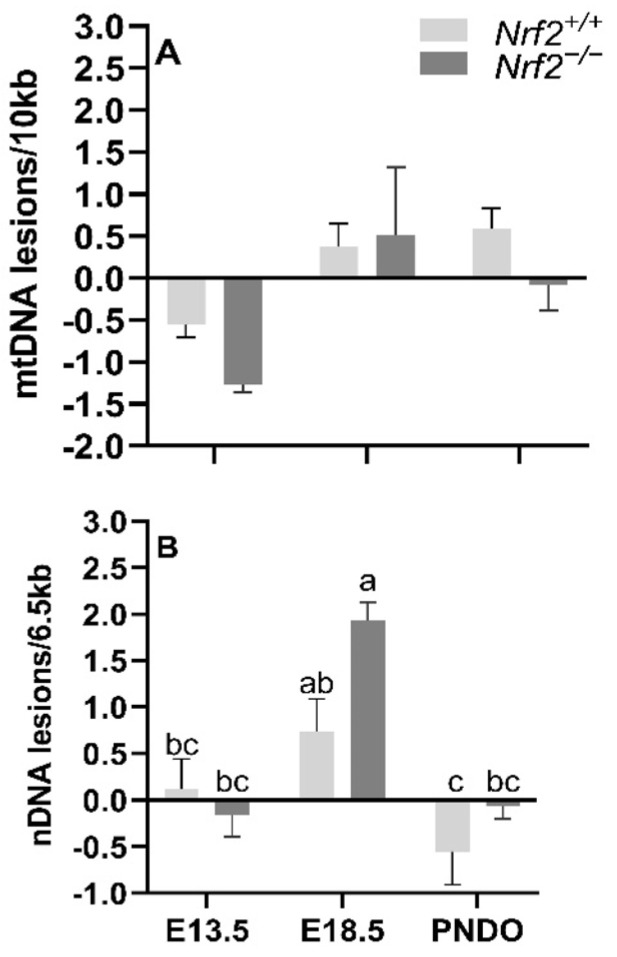
**Effect of *Nrf2* deletion on lung DNA lesions during embryonic development and at birth.** Lesions in mitochondrial (mtDNA, (**A**) and nuclear DNA (nDNA, (**B**) were determined in wild-type (*Nrf2^+/+^*) and *Nrf2*-deficient (*Nrf2^−/−^*) mouse lungs at embryonic (E) days 13.5 and 18.5 or postnatal day 0 (PND0, day of birth) using a gene-specific qPCR assay (lesions per 10 Kb for mtDNA or per 6.5 kb for nDNA). *Nrf2^+/+^* Mean ± S.E.M. presented (*n* = 5–6/group). A two-way ANOVA was employed with age (E13.5, E18.5, PND0) and genotype (*Nrf2^+/+^*, *Nrf2^−/−^*) as the factors. Letters were used when there were significant main effects (*p* < 0.05). Bars not connected by the same letter are significantly different (*p* < 0.05).

mtDNA lesions increase from embryonic day E13.5 to E18.5 independent of NRF2 involvement: We identified a significant main effect of time on mtDNA lesions, where there was a greater accumulation of mtDNA lesions from E13.5 to E18.5 in all embryonic lungs independent of NRF2 involvement (E13.5 mean mtDNA lesions: −0.91 ± 0.24 mtDNA lesions/10 Kb), E18.5 mean mtDNA lesions: 0.44 ± 0.25 mtDNA lesions/10 Kb; *p* = 0.001). We did not find an interaction between the age and mouse model (*p* = 0.40) (Figure 2A).

Nrf2 deficiency leads to increased nDNA lesions from embryonic days E13.5 to E18.5 but recovers at PND0: While we identified a general increase in nDNA accumulations from E13.5 to E18.5, the increase was only significant in *Nrf2^−/−^* embryonic lungs (E13.5: −0.16 ± 0.23 nDNA lesions/6.5 Kb, E18.5: 1.93 ± 0.20 nDNA lesions/6.5 Kb; *p* = 0.01; Figure 2B), but not in *Nrf2^+/+^* mice (E13.5: 0.11 ± 0.33 nDNA lesions/6.5 Kb, E18.5: 0.73 ± 0.36 nDNA lesions/6.5 Kb; *p* = 0.63). From E18.5 to PND0, nDNA lesions significantly decreased in Nrf2^+/+^ (E18.5 embryonic lungs: 0.73 ± 0.36 nDNA lesions/6.5 Kb, PND0 lungs: −0.55 ± 0.36 nDNA lesions/6.5 Kb; *p* = 0.04) and *Nrf2^−/−^* embryonic lungs (E18.5 embryonic lungs: 1.93 ± 0.20 nDNA lesions/6.5 Kb, PND0 lungs: −0.06 ± 0.14 nDNA lesions/6.5 Kb; *p* = 0.001) (Figure 2B).

**Figure 3:**
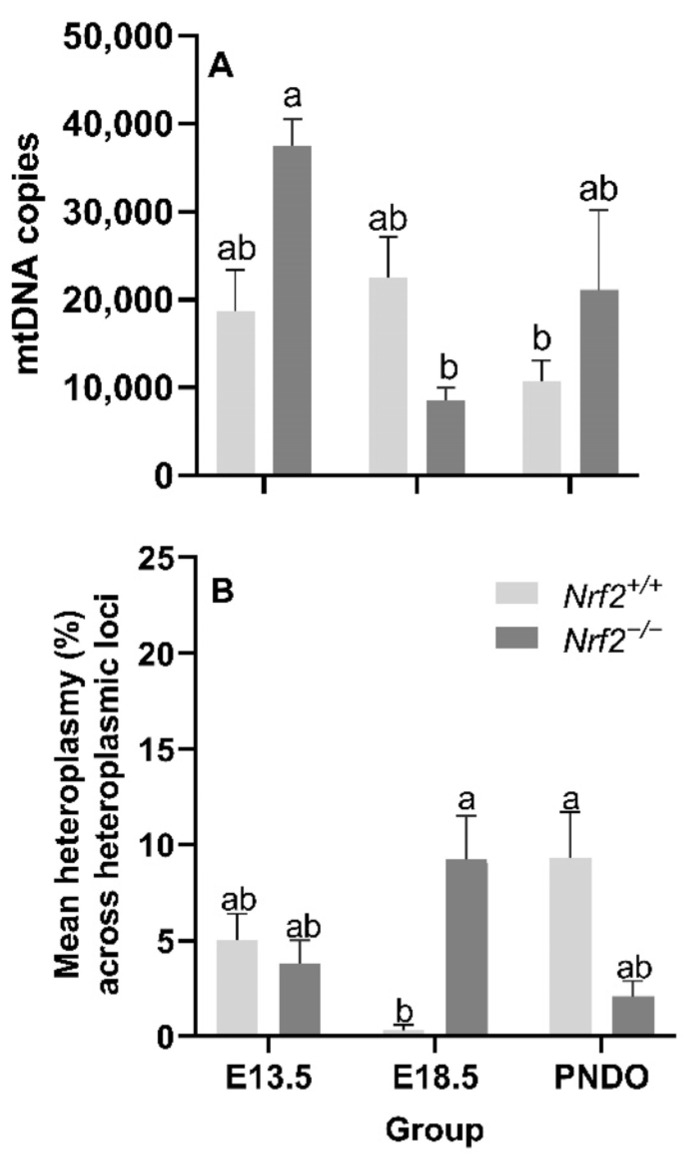
**Effect of *Nrf2* deletion on lung mitochondrial genome diversity during embryonic development and at birth.** (**A**) Mitochondrial DNA copy number (mtDNA copies/3 ng/µL DNA) was determined in wild-type (*Nrf2^+/+^*) and *Nrf2*-deficient (*Nrf2^−/−^*) mouse lungs at embryonic (E) days 13.5 and 18.5 or postnatal day 0 (PND0, day of birth) using a gene-specific qPCR assay. (**B**) Average percent mitochondrial DNA heteroplasmy frequency across heteroplasmic loci in *Nrf2^+/+^* and *Nrf2^−/−^* during embryonic development and birth. *Nrf2^+/+^* at E18.5 (*p* = 0.0003) had the lowest mean heteroplasmy frequency across heteroplasmic loci (INSERT data) compared to all other groups (INSERT data). Mean ± S.E.M. presented (*n* = 5–6/group). A two-way ANOVA was employed with age (E13.5, E18.5, PND0) and genotype (*Nrf2^+/+^*, *Nrf2^−/−^*) as the factors. Letters were used when there were significant main effects (*p* < 0.05). Bars not connected by the same letter are significantly different (*p* < 0.05).

Nrf2 deficiency leads to decreased mtDNA copy numbers from embryonic days E13.5 to E18.5 but recovers at PND0: We did not identify a main effect of age (*p* = 0.05) or mouse model (*p* = 0.27) on the mtDNA copy number; however, we did find a significant interaction effect (*p* = 0.02). *Nrf2^−/−^* embryonic lung mtDNA copies significantly decreased from E13.5 (37,529 ± 3014 mtDNA copies) to E18.5 (8765 ± 1396 mtDNA copies (*p* = 0.02; Figure 3A).

Age and Nrf2 deficiency significantly alter mtDNA heteroplasmy frequency but not the major mtDNA sequence: While we did not find a significant main effect of age (*p* = 0.70) or mouse model (*p* = 0.90) on the mean lung heteroplasmy frequency across heteroplasmic loci, we did observe a significant interaction between the factors *p* = 0.0003). At E18.5, lung heteroplasmy frequency was significantly higher in *Nrf2^−/−^* (9.2 ± 2.3% mean lung heteroplasmy frequency across heteroplasmic loci) versus *Nrf2^+/+^* (0.3 ± 0.3% mean lung heteroplasmy frequency across heteroplasmic loci) (*p* = 0.01). However, at PND0, this effect reversed; the mean lung heteroplasmy frequency was significantly lower in *Nrf2^−/−^* lungs (2.1 ± 0.8% mean lung heteroplasmy frequency across heteroplasmic loci) compared to *Nrf2^+/+^* (9.3 ± 2.4% mean lung heteroplasmy frequency across heteroplasmic loci) (*p* = 0.01) (Figure 3B). We did not identify mtDNA variants or mutations in *Nrf2^−/−^*embryonic lungs or at PND0 (data not shown).

### 3.2. Postnatal Hyperoxia Exposure

**Figure 4:**
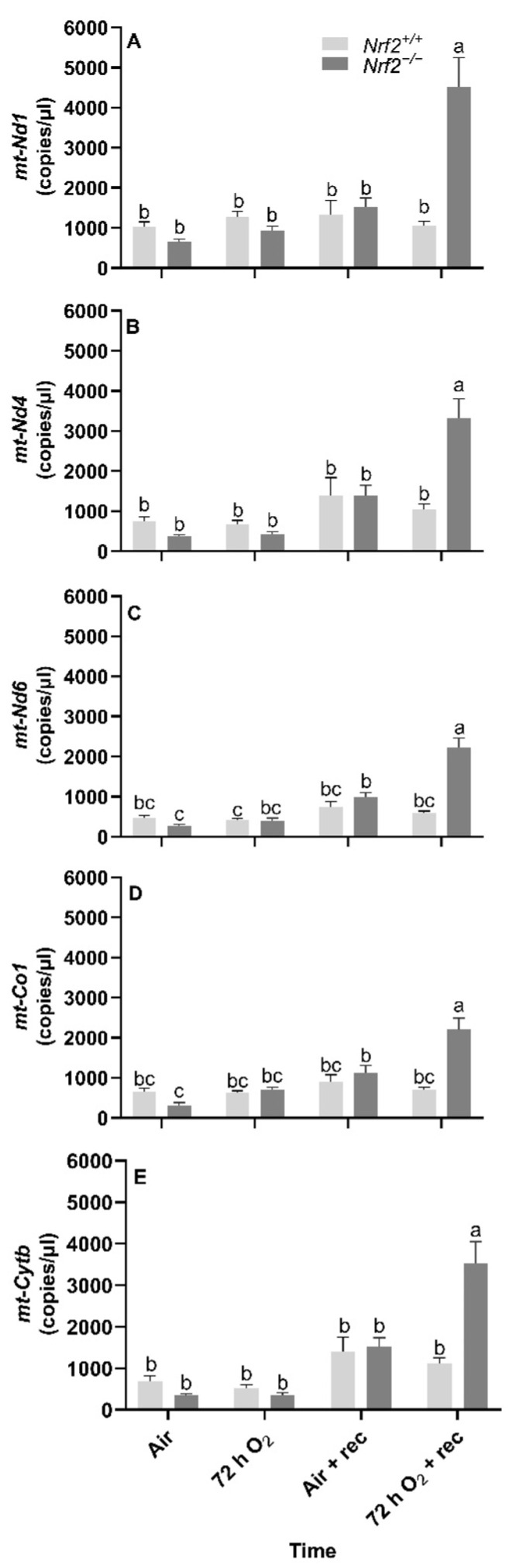
Effect of *Nrf2* deletion on lung mitochondria-encoded mitochondrial gene expression in mice exposed to neonatal hyperoxia. Mitochondria-encoded mitochondrial genes, NADH dehydrogenase 1 (*mt-Nd1*) (**A**), *mt-Nd4* (**B**), *mt-Nd6* (**C**), cytochrome c oxidase subunit 1 (*mt-Co1*) (**D**), and cytochrome B (*mt-Cytb*) (**E**), were determined by digital droplet PCR in lungs of wild-type (*Nrf2^+/+^*) and *Nrf2*-deficient (*Nrf2^−/−^*) mouse neonates exposed neonatally to 72 h room air (air) or 72 h hyperoxia (>95% O_2_,72 h O_2_) from postnatal day (PND) 1 to 4 and the neonatal air- or O_2_-exposed mice recovered in room air for 5 weeks (air + 5 week rec, 72 h O2 + rec). Mean ± S.E.M. presented (*n* = 3–5/group). A two-way ANOVA was employed with genotype (*Nrf2^+/+^*, *Nrf2^−/−^*) and exposure (air, 72 h O_2_, air + rec, 72 h O_2_ + rec) as the factors. Letters were used when there were significant main effects (*p* < 0.05). Bars not connected by the same letter are significantly different (*p* < 0.05).

*Nrf2* deficiency reduces mtDNA-encoded gene expression at 5 weeks following 72 h hyperoxia exposure. Following the day of birth in postnatal *Nrf2*^+/+^ and *Nrf2^−/−^* mice exposed to different extrauterine environments (air or hyperoxia), we found significant overall effects in gene expression in all the mtDNA-encoded genes that we assessed, including *mt-Nd1* (*p* < 0.0001, Figure 4A), *mt-Nd4* (*p* < 0.0001, Figure 4B), *mt-Nd6* (*p* < 0.0001, Figure 4C), *mt-Co1* (*p* < 0.0001, Figure 4D), and *mt-Cytb* (*p* < 0.0001, Figure 4E).

Across all mitochondrial genes that we assessed, we found three- to four-fold increases in gene expression in *Nrf2^−/−^* mice exposed to 72 h hyperoxia following 5 weeks of exposure compared to all other groups.

**Figure 5:**
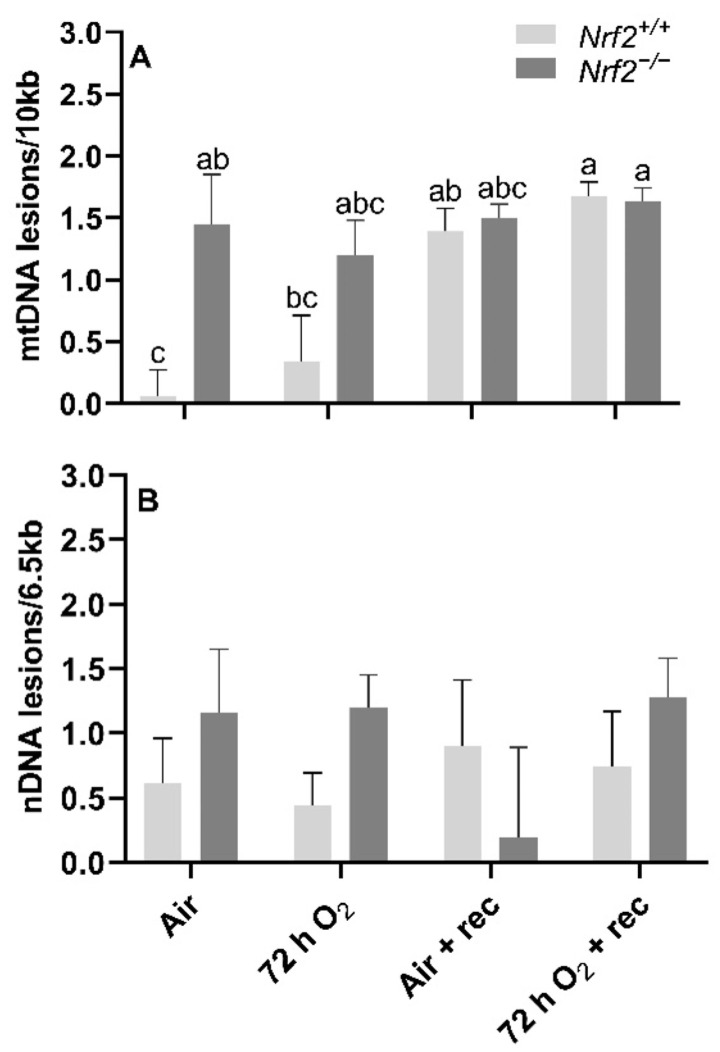
Effect of *Nrf2* deletion on lung DNA lesions in mice exposed to neonatal hyperoxia. recovery. Lesions in lung mitochondrial (mtDNA, (**A**)) and nuclear DNA (nDNA, (**B**)) were determined in mouse neonates exposed to 72 h room air (Air) or 72 h hyperoxia (>95% O_2_,72 h O_2_) from postnatal day (PND) 1 to 4 and the neonatal air- or O_2_-exposed mice recovered in room air for 5 wks (Air + 5 wk rec, 72 h O_2_ + rec) using PCR (lesions per 10 kb for mtDNA or per 6.5 kB for nDNA). Mean ± S.E.M. presented (*n* = 3–5/group). A Two-way ANOVA was employed with genotype (*Nrf2+/+*, *Nrf2^−/−^*) and exposure (Air, 72 h O_2_, Air + rec, 72 h O_2_ + rec) as the factors. Letters were used when there were significant main effects (*p* < 0.05). Bars not connected by the same letter are significantly different (*p* < 0.05).

mtDNA lesions are greater in *Nrf2*-deficient neonates compared to wild type at PND4, though further changes in mtDNA lesions with age and exposure occur independently of Nrf2 involvement. At postnatal day 4, following 72 h O_2_ or air-control exposure, we found that mtDNA lesions were significantly higher in *Nrf2^−/−^* mice (1.20 ± 0.28 mtDNA lesions/10 Kb) compared to *Nrf2^+/+^* (0.44 ± 0.37 mtDNA lesions/10 Kb) in air-controlled conditions (*p* = 0.02). Interestingly, the level of mtDNA damage we found at postnatal day 4 in *Nrf2^−/−^* mice remained at similar levels to the end of the 5-week recovery period irrespective of exposure (*p* = 0.31). However, in *Nrf2^+/+^* mouse lungs only, we observed a trending accumulation of mtDNA lesions with time, and a significant increase noted from postnatal day 4 (0.60 ± 0.21 mtDNA lesions/10 Kb) to the end of the 5-week recovery period (0.90 ± 0.19 mtDNA lesions/10 Kb) (*p* = 0.047), independent of exposure (Figure 5A).

**Figure 6:**
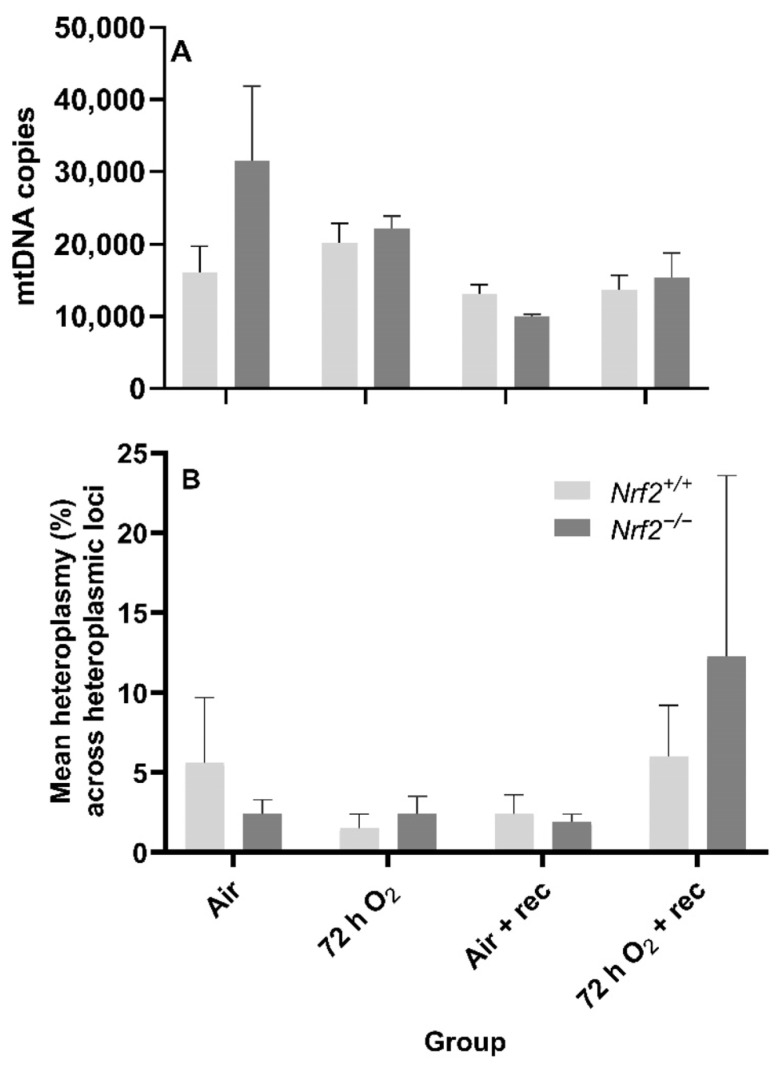
Effect of *Nrf2* deletion on lung mitochondrial genome diversity in mice exposed to neonatal hyperoxia. (**A**) Lung mitochondrial DNA copy number (mtDNA copies/3ng/µl DNA) was determined in mouse neonates exposed to 72 h room air (Air) or 72 h hyperoxia (>95% O_2_,72 h O_2_) from postnatal day (PND) 1 to 4 and the neonatal air- or O_2_-exposed mice recovered in room air for 5 wks (Air + 5 wk rec, 72 h O_2_ + rec) by a gene specific qPCR assay. (**B**) Average percent mitochondrial DNA heteroplasmy frequency across heteroplasmic loci in *Nrf2+/+* and *Nrf2^−/−^* mice. Heteroplasmic loci = mtDNA position where the minor allele occurrence ≥ 1% (i.e., heteroplasmy). A Two-way ANOVA was employed with genotype (*Nrf2+/+*, *Nrf2^−/−^*) and exposure (Air, 72 h O_2_, Air + rec, 72 hr O_2_ + rec) as the factors. Letters were used when there were significant main effects (*p* < 0.05). Bars not connected by the same letter are significantly different (*p* < 0.05). Mean ± S.E.M. presented (*n* = 3–5/group). No significant difference in the mean percent heteroplasmy frequency across heteroplasmic loci across groups (*p* = 0.97).

Nrf2 deficiency nor 72 h hyperoxia exposure influence nDNA lesions or mtDNA copy number. We did not observe significant differences between *Nrf2*-deficient or wild-type mice in nDNA lesions (*p* = 0.49; Figure 5B) or mtDNA copy number (*p* = 0.11; Figure 6A) according to exposure or age.

Neither Nrf2 deficiency nor 72 h hyperoxia exposure influence the onset of de novo mtDNA variants or heteroplasmy. While we expected mtDNA heteroplasmy frequency would be altered during these postnatal stages or due to 72 h O_2_ exposure, we did not find significant differences between the heteroplasmy frequency of *Nrf2*-deficient or wild-type mice based on exposure (*p* = 0.84) or with age (*p* = 0.55) (Figure 6B). We also did not identify the onset of de novo mtDNA variants or mutations in any *Nrf2^−/−^* mice (data not shown).

## 4. Discussion

Prior work from our group demonstrated the critical role of the NRF2 in mediating protection against oxidant-induced lung injury in mouse neonates exposed to hyperoxia [6]. However, until now, the role of NRF2 on mtDNA-encoded gene expression—as well as its genome components—had not been investigated. In the current work, we sought to assess the role of NRF2 on aspects of the mitochondrial genome during embryonic development and postnatally, with 72 h O_2_ exposure in mice to mimic human preterm-birth oxidant stress with supplemental O_2_.

### 4.1. Role of Nrf2 in Lung Mitochondrial Genome and DNA Lesions during Embryonic Development to the Day of Birth

Because mtDNA is highly susceptible to damage when exposed to oxidative stress, given its proximity to the ETC [10], oxidant stressors can significantly alter mtDNA integrity, copy number, and heteroplasmy. Under rare circumstances, de novo mtDNA variants and/or mutations can arise.

During stages E13.5 and E18.5 to PND0, we assessed the effect of NRF2 on multiple aspects of the mitochondrial genome, including gene expression in five of the 13 mtDNA-encoded genes, heteroplasmy, copy number and integrity (mitochondrial and nuclear DNA lesions). Briefly, the time between E13.5 and birth is when significant shifts in metabolic metabolism occur [15]. In mice cardiac cells, by E13.5, mitochondria are considered “almost mature” (class 3) [15]. There is a metabolic shift between E13.5 and the end of gestation from heavy reliance on glycolysis for ATP production to aerobic metabolism in mice. Because of NRF2’s known role in mediating antioxidant responses with oxidative stress, we hypothesized that NRF2 deficiency would lead to greater accumulations in lung DNA lesions with resulting reductions in mtDNA copy number and increased heteroplasmy frequency.

Further, given that the mitochondrial genes we included reflect oxidative phosphorylation, we expected decreased gene expression in *Nrf2^−/−^* mice at PND0, when ATP production is primarily reliant on oxidative metabolism. We observed several notable changes in some of the parameters in which NRF2 deficiency had a significant effect. In contrast, others occurred independently of NRF2, which likely reflects the ongoing metabolic shift from the embryo to birth.

To our surprise, we did not identify changes in expression of any of the mtDNA-encoded genes, suggesting that regulation of mitochondrial maturation in response to metabolic shift arises from nuclear-encoded mitochondrial genes. Furthermore, while *mt-Nd4* expression significantly decreased in *Nrf2^−/−^* embryonic lungs from E13.5 to E18.5, the change was similar between *Nrf2^−/−^* and *Nrf2^+/+^* at E18.5 and PND0, indicating an insignificant role of NRF2 in *mt-Nd4* transcription.

For DNA lesions, we did not find changes in accumulated lung mtDNA lesions with time or due to NRF2, though we did identify significant differences in nuclear DNA (nDNA) lesions, mtDNA copy number, and heteroplasmy. Changes in nDNA lesions occurred independently of NRF2. The greatest nDNA lesions accumulations occurred at E18.5. It is unclear why nDNA lesions accumulate at this time, though nDNA lesions are considered an indirect marker of oxidative stress. As such, this late stage of embryonic development is likely presented with high ROS production that incurs significant nDNA lesions. Because significant mitochondrial maturation occurs during embryonic development, we initially thought mtDNA lesions would change with time and in the absence of NRF2. However, mitochondrial maturation is tightly regulated during embryonic development, and damaged/dysfunctional mitochondria are quickly degraded and removed from the embryo under normal circumstances [24]. Thus, it is possible that this mitochondrial fine-tuning allows for only functional mitochondria to remain, where their DNA components are unaffected. To indirectly support this fine-tuning thought, we noted significant changes in mtDNA copies in *Nrf2^−/−^* embryonic lungs only from E13.5 to E18.5. The lack of NRF2 protection in these mice during a time of significant metabolic shifting potentially reflects mitochondrial mitophagy and ongoing fission and fusion to maintain healthy mitochondria.

Alongside our initial expectation that NRF2 deficiency would lead to accumulated mtDNA lesions during embryonic development and PND0 compared to the wild type, we also suspected we would identify de novo mtDNA variants (i.e., mtDNA variants not found in the mother and/or that developed with time), decreased mtDNA copies, and alterations in heteroplasmy. While we did not identify mtDNA variants (data not shown), we did observe that *Nrf2^−/−^* heteroplasmy frequency was significantly higher than *Nrf2^+/+^* at E18.5, though this effect reversed at PND0.

While recent technological advances in ultra-deep DNA sequencing have enabled detection of heteroplasmy, the within and between cell variation in mtDNA heteroplasmy frequency continues to present challenges in linking the presence of heteroplasmy with phenotypes. Given this known variability in heteroplasmy with current techniques, we analyzed heteroplasmy in this current study by comparing the mean percent heteroplasmy across heteroplasmic loci. Additionally, our findings include site-specific heteroplasmy (Supplementary Data File S1), though the heteroplasmy variability by loci and gene inhibited running a statistical analysis. However, we observed some important, statistically significant findings and other notable trends. First, though statistically insignificant in *Nrf2^−/−^*mice, heteroplasmy trended upward from E13.5 to E18, then decreased at PND0.

Interestingly, these trends in heteroplasmy frequency were opposite in *Nrf2^+/+^* mice, where there was a trending decrease from E13.5 to E18.5, then a significant increase to PND0. While it is beyond the scope of this current work, our group has hypothesized that increased heteroplasmy frequency—under the assumed disease threshold of 60–80%—increases an organism’s ability to respond to oxidative stress stimuli by creating greater genetic diversity. From these current findings, we cannot conclude the mechanism through which NRF2 influences lung heteroplasmy, though we believe it is important to note the decreased heteroplasmy in *Nrf2^−/−^*-deficient mice at PND0. Some loci that we identified with heteroplasmy (Supplementary Data File S1) occur at regions that influence mtDNA replication, ribosomal, and/or oxidative phosphorylation. Small changes in the mitochondrial genome can significantly affect mitochondrial function. Thus, heteroplasmy occurrence at such loci regulating functions critical for mitochondrial architecture, oxidative capacity, and its DNA replication should be considered in future work.

### 4.2. Role of NRF2 on Lung Mitochondrial Genome and DNA Lesions in Neonatal Mice Exposed to Hyperoxia

In our second aim of this study, we assessed the effect of NRF2 on the same lung-mitochondrial genomic parameters and DNA lesions, as in aim 1, in postnatal mice exposed to 72 h hyperoxia and following a 5-week recovery period.

We found *Nrf2^−/−^* mice had three- to four-fold increased gene expression in all mitochondrial genes we assessed five weeks after hyperoxia exposure compared to all other groups and time points. Thus, these findings strongly suggest that NRF2 influences lung mitochondrial oxidative phosphorylation pathways in neonates exposed to hyperoxia, at least partially via modulating mtDNA-encoded mitochondrial proteins. While our data cannot directly conclude that NRF2 also influenced mitochondrial function (e.g., oxidative capacity) with hyperoxia exposure, we found significant increases in mitochondrial genes that regulate components involved in oxidative phosphorylation. This indicates that oxidative capacity might be increased in *Nrf2*-deficient mice in the weeks following hyperoxia. The increased mitochondrial gene expression contradicts our original hypothesis that expression in these genes would decrease with *Nrf2* deficiency. However, some findings in other work support compensatory increased mitochondrial oxygen consumption in adults born preterm [25] and in diseased states, such as in individuals with Type 2 diabetes [26]. Thus, the increased gene expression in mitochondrial genes that inform oxidative phosphorylation may reflect compensatory increased mitochondrial oxygen consumption in the weeks following hyperoxia exposure in an *Nrf2*-deficient state. Importantly, we show an influence of NRF2 on mitochondrial genes that regulate oxidative phosphorylation pathways that may have significant implications during postnatal development and early disease onset.

We also assessed mtDNA variants for heteroplasmy, copy number, and DNA lesions (mitochondrial and nuclear) in this aim. We did not identify mtDNA variants or significant changes with time or NRF2 in any of these parameters. However, in mtDNA lesions only, we found that *Nrf2^−/−^* neonates had significantly greater mtDNA damage than *Nrf2^+/+^*, though the lesions that accumulated following this time point were similar between *Nrf2^+/+^* and *Nrf2^−/−^* mice, regardless of exposure type.

Our most notable finding in this aim was the significant increases in mtDNA-encoded gene expression in the weeks following hyperoxia exposure in *Nrf2^−/−^* mice. This highlights the importance of future work to investigate long-term effects of NRF2 on lung mitochondrial function and its link with susceptibility to lung injury (e.g., bronchopulmonary dysplasia).

Our study has a few limitations related to its translation into the role of NRF2 in human lung mitochondrial maturation during embryonic development and, postnatally, when neonates are first exposed to air as an oxidant stressor or with supplemental O_2_. Given the very small lungs and the inability to isolate an adequate number of specific types for analyses used in our work, we only assessed whole lung homogenates. Because mitochondrial genomic parameters, such as heteroplasmy and copy number, are highly variable across cell and tissue types, it is possible that having an ability to assess different cell types within the lung would have yielded less variable data within groups and potentially cell-type-specific loci within the mitochondrial genome affected by heteroplasmy or copy-number changes. Further, our findings would have benefited from complementary lung histology, transmission electron microscopy, and inflammatory markers to inform lung injury and future disease risk in these mice. Multiple studies indicate that in humans born preterm, it is linked with the early life onset of chronic pulmonary diseases (e.g., bronchopulmonary dysplasia) and cardiometabolic conditions [27,28]. Thus, future work must investigate the role of NRF2 with the onset of early life chronic conditions in individuals born preterm. However, in this current investigation, our primary goal was to determine if NRF2 affected lung mitochondrial function and genomic characteristics during embryonic development and early postnatally, which required a substantial number of mice. Importantly, our findings lay the foundation for the next steps in understanding the link between NRF2 and mitochondrial function with mitochondrial maturation in the womb and lung-injury protection at birth. In this way, the last limitation of this current work is that we could not directly assess mitochondrial function and/or ROS production.

In summary, our findings highlight that NRF2 may have an important role in mediating supplemental O_2_-induced oxidative stress on mitochondrial function during postnatal development. While it is unclear whether increased mtDNA-encoded gene expression align with increased mitochondrial oxygen consumption in the weeks following hyperoxia exposure, other work in adults born preterm exhibited excessive mitochondrial oxygen consumption [25]. Given that NRF2 controls mitochondrial ROS production, and our data suggest mitochondrial oxidative capacity is altered within weeks following hyperoxia exposure, this emphasizes a critical need to determine the influence of NRF2 on mitochondrial dysfunction and early onset of cardiopulmonary diseases in individuals born preterm. Though we initially thought NRF2 would have a role in altering mitochondrial genomic characteristics during embryonic lung development and postnatally, we did not identify significant alterations, suggesting NRF2 changes in mitochondrial genome transcription occurs through nuclear involvement only. Future studies are needed to assess NRF2′s role in specific lung mitochondrial function parameters (e.g., oxidative phosphorylation subunit activity, membrane potential, ROS production, etc.) and its proteome to begin developments towards using NRF2 as a molecular target to offset mitochondrial dysfunction when hyperoxia is exposed at birth; thus preventing lung tissue injury and aiding normal postnatal development.

With continued work on preterm birth and the role of NRF2 as a mechanistic target to prevent oxidative-stress-induced mitochondria and, ultimately, tissue injury, there are several important clinical applications and implications included within this topic. The observation that NRF2 deficiency results in decreased mitochondria-encoded mitochondrial gene expression following a 5-week recovery period from hyperoxia exposure, but not acutely, leaves the important clinical questions:

1. Do these gene-expression values reflect impaired mitochondrial function, and, if so, how long do these mitochondrial impairments last?
2. What are the effects on other tissues and cell types?
3. Could a nutritional and/or therapeutic modality targeting enhanced antioxidant capacity via NRF2 early in life (i.e., at birth) be used as an intervention?

Addressing these clinically relevant questions is critical. Our current findings suggest mitochondria, at least in the lung, are adversely affected in mice at an age equivalent to human puberty/early adolescent years. Impaired mitochondrial function can lead to numerous cardiometabolic conditions [29,30] and some forms of cancer [31], thus contributing to disproportionately higher disease risk of other diseases early in life in this population. In another recently published work by our group, we found that postnatal hyperoxia exposure led to increased susceptibility to the Respiratory Syncytial Virus and Bronchopulmonary dysplasia symptoms in young adult mice, indicating preterm birth has significant adverse effects on health span [5]. Additionally, in humans, young adults born preterm have a greater incidence of cardiovascular-disease-related events and deaths early in life [29,30]. Thus, developing and establishing early life interventions to prevent oxidative stress-induced mitochondrial dysfunction and tissue impairments are critical for the overall health span of individuals born preterm. Further, clinical interventions and future work are also needed to address health outcomes in children, adolescents, and adult survivors of preterm birth.

## Figures and Tables

**Table 1:** Mitochondria Encoded Mitochondrial Gene Information.

Gene Symbol	Assay ID	Probe Fluorophore	Species
ND1	dMmuCNS343824284	FAM	Mouse
ND4	dMmuCNS709202118	FAM	Mouse
ND6	dMmuCNS680369013	FAM	Mouse
COX1	dMmuCNS882365391	HEX	Mouse
CYTB	dMmuCNS387155035	HEX	Mouse
Rpl13a	dMmuCPE5091711	HEX	Mouse

**Table 2:** Mouse Gene Targets and Primer Pairs for QPCR.

6.5-kb Fragment from the β-Pol Gene, Accession Number, AA79582		
MBFor1	5′-TAT CTC TCT TCC TCT TCA CTT CTC CCC TGG-3′	Sense
MBEX1B	5′-CGT GAT GCC GCC GTT GAG GGT CTC CTG-3′	Antisense
10-kb mitochondria fragment		
2372	5′-GCC AGC CTG ACC CAT AGC CAT ATT AT-3′	Sense
13,337	5′-GAG AGA TTT TAT GGG TGT ATT GCG G-3′	Antisense
117-bp mitochondria fragment		
13,597	5′-CCC AGC TAC TAC CAT CAT TCA AGT-3′	Sense
13,688	5′-GAT GGT TTG GGA GAT TGG TTG ATG-3′	Antisense

## Data Availability

Data are contained within the article or Supplementary Material.

## Supplementary Materials

The following supporting information can be downloaded at: https://www.mdpi.com/article/10.3390/antiox11040760/s1. Supplementary Data File S1.
